# CLPB deficiency-associated congenital neutropenia: A rare case report and literature review

**DOI:** 10.1097/MD.0000000000046987

**Published:** 2026-01-16

**Authors:** Yuxing Sun, Min Tang, Juan He, Xiaoqin Hu, Ming Li, Li Tan

**Affiliations:** a Department of Respiration, Kunming Children’s Hospital, Kunming, Yunnan Province, China.

**Keywords:** 3-methylglutaconic acid, CLPB mutation, neurologic disorder, neutropenia

## Abstract

**Rationale:**

Congenital neutropenia (CN) encompasses a group of disorders characterized by impaired neutrophil differentiation, resulting in persistently low neutrophil counts in the peripheral blood. It presents with recurrent infections and an elevated risk of leukemia. Multiple genetic mutations have been implicated in the pathogenesis of neutropenia.

**Patient concerns:**

This paper reports the case of a 3-year-2-month-old boy admitted with a 4-day history of cough and fever, accompanied by recurrent respiratory infections, neutropenia, and growth retardation. Whole-exome sequencing identified a mutation in the caseinolytic peptidase B homolog (CLPB) gene (NM_030813.6: c.1681C>T: p.R561W).

**Diagnoses:**

Although the initial genetic sequencing did not reveal mutations consistent with the clinical presentation, the child continued to experience recurrent infections. Upon reanalysis, a pathogenic CLPB-related mutation was detected, leading to the diagnosis of CN.

**Interventions:**

During hospitalization, the patient received targeted antimicrobial therapy based on the identification of the pathogen. Following the confirmed diagnosis, he also received intermittent granulocyte colony-stimulating factor therapy.

**Outcomes:**

Administration of granulocyte colony-stimulating factor successfully maintained neutrophil counts above 0.5 × 10^9^/L and significantly reduced the frequency of respiratory tract infections.

**Lessons:**

CLPB deficiency should be considered in pediatric patients presenting with CN and concurrent neurological symptoms, as early recognition allows for the timely initiation of appropriate treatment strategies and contributes to improved clinical outcomes.

## 1. Introduction

Congenital neutropenia represents a heterogeneous group of genetic disorders that disrupt neutrophil production, differentiation and survival within the bone marrow, leading to recurrent infections and an increased risk of progression to myelodysplastic syndromes or acute myeloid leukemia. The generally accepted threshold for absolute neutrophil count (ANC) is 1.0 × 10^9^/L in infants up to 1 year of age and 1.5 × 10^9^/L from 1 year through adulthood. However, these values may vary across different ethnic populations.^[1]^ The likelihood and prognosis of bacterial infections associated with neutropenia depend not only on the peripheral blood ANC but also on the capacity of neutrophils to migrate effectively to sites of infection.^[2]^ Agranulocytosis, a severe form of neutropenia, defined by an ANC below <0.2 × 10^9^/L, carries a significantly increased risk of life-threatening infections.^[3]^ Gene mutations, particularly in the *ELANE, GFI1, HAX1, G6PC3, VPS45, JAGN1, CSF3R, SRP54,* and *WAS* genes, are associated with severe congenital neutropenia (SCN).^[4]^ In 2015, researchers identified loss-of-function mutations in the caseinolytic peptidase B homolog (CLPB) gene, which encodes CLPB, as a cause of a distinct phenotype characterized by 3-methylglutaconic aciduria, neurological impairment, neutropenia, cataracts, and high early mortality.^[5–8]^

However, the clinical presentation of CLPB mutations can be variable; while not all patients show the full spectrum of features, neutropenia is frequently observed, particularly in those carrying heterozygous variants.^[9]^ More recent studies have further noted premature ovarian failure and infertility in patients who survived beyond puberty.^[10]^ These findings suggest that CLPB variants exert phenotypes effects that differ depending on their genomic location and dosage.^[11]^

In the present study, we present the exome sequencing findings of a child with neutropenia, describe his clinical manifestations and treatment response, and review the relevant literature.

## 2. Case report

The proband, a male toddler, was the second child born to consanguineous parents. Delivery was by cesarean section atr 34 + 4 weeks of gestation due to premature rupture of membranes and a scarred uterus. His birth weight was 2.35 kg, and no evidence of asphyxia. The Apgar score was not available. He remained hospitalized in the maternity hospital for over 1 month because of neonatal pneumonia, prematurity, and low birth weight. During this period, he required invasive ventilator-assisted therapy, though the family reported no prior family history of oxygen therapy.

**Figure 1.**
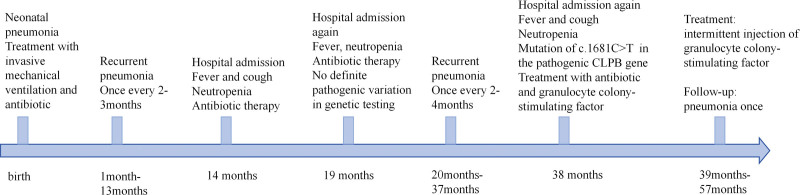
The patient’s condition progressed and related clinical symptoms. CLPB = caseinolytic peptidase B homolog.

Growth and developmental milestones were delayed. The child was able to lift his head at 5 months, sit independently at 15 months, and walked independently at 2 years. At 3 years and 2 months, he could walk approximately 100 meters without assistance but fell frequently, was unable to run, and required help with dressing. He vocalized sounds but had no intelligible speech, displayed poor cognitive function, and showed limited self-care skills. The clinical course and progression of his symptoms are outlined in Figure 1.

At 14 months of age, the child presented to our hospital with a history of persistent fever for 20 days and cough for 14 days, and he was admitted to the intensive care unit. Laboratory tests revealed neutropenia, with a neutrophil count of 0.18 × 10^9^/L. Imaging demonstrated hepatomegaly, bilateral pneumonia, and cavity lesions in the upper lobe of the right lung and the lower lobe of the left lung. Brain magnetic resonance imaging (MRI) revealed deepened and widened cerebral sulci, widened bilateral frontotemporal extracerebral space, and enlargement of both lateral ventricle and the third ventricle. Microbiological evaluation identified *Streptococcus* and *Mycoplasma pneumoniae* as pathogens. Treatment consisted of vancomycin, cefoperazone-sulbactam, and azithromycin. Following clinical improvement, the patient was discharged.

Five months later, he was readmitted with a 4-day history of fever. Investigations again demonstrated neutropenia, with a nadir of 0.31 × 10^9^/L neutrophils. Immunoglobulin assays showed significantly elevated immunoglobulin M, immunoglobulin A , and especially immunoglobulin G (IgG; 29.25 g/L). An immune deficiency was suspected, and targeted treatment was initiated, leading to a reduction in IgG levels. However, chest computed tomography revealed a pulmonary cavity in the left lower lobe, with subsequent progression. Tracheoscopy with alveolar lavage identified *Streptococcus pneumoniae*. The patient was treated with meropenem and vancomycin. After symptoms relief, he was discharged. Genetic testing at that time did not identify any pathogenic variant consistent with his phenotype.

At 3 years and 2 months, the child presented for a third time with cough and fever of 4 days’ duration. Chest computed tomography showed bilateral pneumonia, consolidation, interstitial involvement of the right lung, and cavitary lesions. The ANC was critically low at 0.12 × 10^9^/L, and IgG was further elevated to 43.69 g/L. Ultrasound confirmed a significant hepatomegaly with diffusely enhanced parenchymal echogenicity. Bone marrow aspiration revealed severely reduced granulopoiesis with compensatory erythroid hyperplasia. The patient was treated with cefoperazone-sulbactam and vancomycin, and granulocyte colony-stimulating factor (G-CSF) was administered, successfully maintaining neutrophil counts at approximately 0.5 × 10^9^/L. Clinical status improved under this regimen.

**Figure 2.**
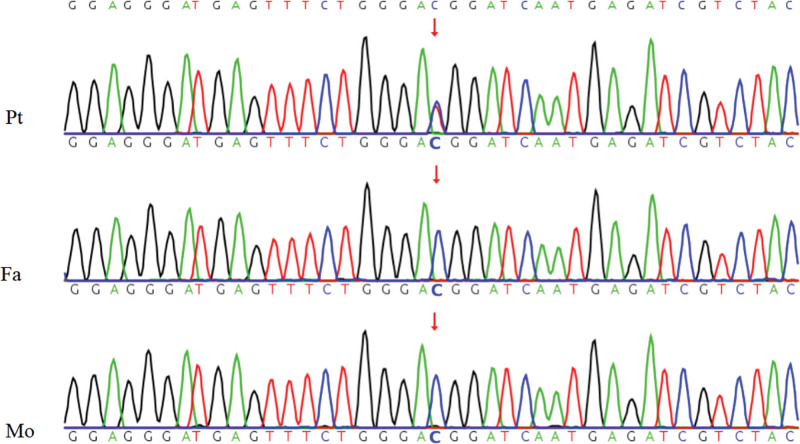
Sanger sequencing of the CLPB gene of the patient (Pt) and close relatives. The patient carried a c.1681C>T (p.R561W) mutation, while his father (Fa) and mother (Mo) harbored the wild type genotype. The patient’s mutation was therefore a spontaneous missense mutation. CLPB = caseinolytic peptidase B homolog.

Eurological evaluation by MRI revealed blurred gray–white matter differentiation, abnormal white matter signal, a significantly thinned corpus callosum, elevated intracranial pressure, and pronounced widening of cerebral sulci, as well as ventricular enlargement. Cardiac ultrasound and ophthalmologic examination revealed no abnormalities. Subsequent whole-exome sequencing identified a pathogenic missense mutation in the CLPB gene (c.1681C>T), which was confirmed by segregation analysis as a de novo event (Fig. 2).

## 3. Discussion

Genetic abnormalities affecting hematopoiesis also impair the development of neutrophil granulocyte, contributing to the pathogenesis of SCN. This condition can progress to agranulocytosis, particularly during infancy, and is characterized by recurrent, severe and potentially life-threatening infections.^[12]^ In 2015, biallelic CLPB mutations were first associated with a constellation of phenotypes, including neurological impairment, neutropenia, 3-methylglutaconic aciduria, and cataracts.^[5,6,8]^ Subsequently, myeloid malignancies have also been reported in patients harboring CLPB mutations in both autosomal dominant and autosomal recessive states.^[4]^ Long-term follow-up of individuals surviving into adulthood has revealed additional postpubertal manifestations, such as premature ovarian insufficiency and infertility.^[10]^

The CLPB gene encodes CLPB, a member of the AAA+ ATPase family that is implicated in multiple cellular processes.^[13]^ Proteomic analyses have demonstrated that CLPB is highly enriched in mitochondria across various tissues, particularly in the brain, and its depletion has been associated with severe neonatal encephalopathy.^[6]^ Although the precise cellular role of CLPB remains unclear, evidence suggests that it is involved in mitochondrial protein folding,^[7]^ the regulation of apoptosis, and the maintenance of cristae structure.^[14]^ To better elucidate the CLPB significance in cells, Capo-Chichi et al^[6]^ used antisense morpholino oligonucleotides to inhibit translation of the zebrafish CLPB orthologue. They demonstrated abnormal responses, including increased swim velocity and tail-beat frequency. These findings mirrored the neuronal hyperexcitability observed in patients with CLPB deficiency. Furthermore, CLPB gene knockdown decreased the population of inhibitory glycinergic interneurons while increasing population of excitatory glutamatergic neuronal, supporting the hypothesis that CLPB functions as a mitochondrial chaperone critical for neuronal regulation.

The clinical spectrum of CLPB deficiency varies in relation to disease severity. Patients with mild deficiency typically show no neurological involvement and maintain normal intellectual development. Moderate deficiency is associated with neurological abnormalities during infancy, including hypotonia, feeding difficulties, progressive motor disorders (ataxia, dystonia, and/or dyskinesia), spasticity, epilepsy, and intellectual disability. Neutropenia in these patients is variable but usually not life-threatening. By contrast, severe deficiency often leads to death within months of birth due to profound neurological dysfunction manifesting as hyperekplexia, hypotonia or hypertonia, absence of voluntary movements, swallowing difficulties, respiratory insufficiency, and epilepsy combined with recurrent, fatal infections.

Our patient presented with craniofacial anomalies, including frontal bossing, hypertelorism, micrognathia, and low-set, posteriorly rotated ears, which have been previously documented in CLPB-deficient infants,^[15]^ although these findings are pathognomonic. A study involving 31 patients from 18 families revealed a strong correlation between the age of onset and severity of clinical presentation. Earlier onset was associated with more severe manifestations, and survival was closely tied to the disease phenotype. The longest survival among mildly affected patients was 25 years, whereas those with moderate disease lived up to 20 years. Nearly half of all reported patients died, and all individuals with severe disease succumbed before the age of 4. Genotype-phenotype correlations have also been described: nonsense and frameshift variants are predominantly associated with severe phenotypes, while missense mutations are more commonly linked to milder disease. CLPB deficiency may also arise from heterozygous variants with autosomal dominant inheritance, whereas biallelic variants follow an autosomal recessive pattern.^[16]^

To date, he Human Gene Mutation Database (http://www.hgmd.org) has cataloged 39 CLPB variants, most of which are missense variants. The c.1681C>T (p.R561W) mutation in exon 15, which replaces arginine with tryptophan at position 561, has been previously described.^[17]^ However, its clinical significance and prognosis remains incompletely understood. In our patient carrying this variant, we observed growth retardation, dysmorphic features, recurrent necrotizing pneumonia with neutropenia, and movement disorders. MRI demonstrated abnormal white matter development consistent with CLPB deficiency. Interestingly, no cataracts were detected, possibly due to the young age at presentation. Although earlier studies^[18]^ have shown elevated urinary excretion of 3-methylglutaconic acid in nearly all CLPB-deficient children, we were unable to confirm this finding in our case due to parental refusal of tandem mass spectrometry testing.

No standard therapy currently exists for CLPB deficiency, and management remains supportive.^[16]^ No specific dietary or metabolic interventions have been validated. Subcutaneous administration of G-CSF has been shown to improve neutrophil counts and decrease the frequency of infections, though responsiveness may vary. Our patient did not receive continuous G-CSF treatment; instead, his parents sought care at local hospital for intermittent injections when neutrophil counts fell blow 0.5 × 10^9^/L, typically lasting 1 to 3 months. G-CSF therapy successfully maintained his neutrophil counts above 0.5 × 10^9^/L and reduced the frequency of infections. For patients with SCN who do not show neurological involvement, hematopoietic stem cell transplantation remains the recommended curative approach. Furthermore, active rehabilitation is essential to optimize motor function and prevent secondary orthopedic complications, such as contracture, scoliosis, and hip dislocation.^[16]^

In conclusion, further investigations are warranted to clarify the etiology of congenital neutropenia, which is often accompanied by recurrent respiratory infections and central nervous system involvement in children. Whole-exome sequencing combined with urinary organic acid analysis offers valuable diagnostic evidence for inherited metabolic disorders.

## Author contributions

**Conceptualization:** Li Tan.

**Data curation:** Juan He.

**Funding acquisition:** Li Tan.

**Resources:** Ming Li.

**Writing – original draft:** Min Tang.

**Writing – review & editing:** Yuxing Sun, Xiaoqin Hu.
